# Modification of Silver-Loaded Biodegradable Polymer Nanoparticles with Bacterial Membrane Vesicles for Treating Intracellular Bacterial Infections

**DOI:** 10.3390/ma18153470

**Published:** 2025-07-24

**Authors:** Wei Xu, Sayo Maruyama, Takuro Niidome

**Affiliations:** 1 Faculty of Advanced Science and Technology, Kumamoto University, 2-39-1 Kurokami, Chuo-ku, Kumamoto 860-8555, Japan; 231d8772@st.kumamoto-u.ac.jp (S.M.); niidome@kumamoto-u.ac.jp (T.N.); 2 Institute of Industrial Nanomaterials, Kumamoto University, 2-39-1 Kurokami, Chuo-ku, Kumamoto 860-8555, Japan

**Keywords:** silver nanoparticles, membrane vesicles, intracellular bacteria, macrophage

## Abstract

*Salmonella enterica* serovar Typhimurium (*S*. *Typhimurium*) is an intracellular pathogen capable of surviving and replicating within macrophages, which causes foodborne diseases such as gastroenteritis. To develop a strategy against intracellular bacteria in macrophages, we designed silver-loaded biodegradable polymer nanoparticles functionalized with *S*. *Typhimurium* membrane vesicles (MVs). Silver nanoparticles (Ag NPs) were initially encapsulated within biodegradable poly(lactic-co-glycolic) nanoparticles (Ag-P NPs), which were then surface-modified with polyethyleneimine to form Ag-PP NPs. These were subsequently fused with *S. Typhimurium* MVs via a sonication method to generate Ag-PP@MV NPs. The resulting MV-coated nanoparticles displayed a similar protein profile to that of native MVs and exhibited antibacterial activity against intracellular *S*. *Typhimurium*. Notably, the enhanced cellular uptake of the MV-modified NPs contributed to their intracellular bactericidal efficacy. This study highlights MV modification as a promising strategy to improve NP delivery to macrophages for treating persistent intracellular infections.

## 1. Introduction

Phagocytes, such as macrophages, neutrophils and dendritic cells, play a central role in the innate immune response through their unique ability to internalize and destroy microorganisms [1]. However, specific intracellular pathogens, such as *Salmonella enterica* serotype Typhimurium (*S*. *Typhimurium*), evade this defense by forming Salmonella-containing vacuoles (SCVs) within macrophages, thereby avoiding host-mediated destruction [2,3]. *S. Typhimurium* is a major cause of foodborne illnesses, including gastroenteritis. Although various antibiotics are clinically used to treat intracellular bacterial infections, achieving complete eradication of such infections is challenging. Moreover, the rise in antibiotic-resistant strains further complicates treatment and poses a serious public health threat [4]. Strains such as sequence types 313 (ST313) and ST34 of *S*. *Typhimurium* lineages are associated with invasive diseases in parts of Africa and Southeast Asia, respectively, and they have independently developed resistance to the antibiotic ciprofloxacin [5].

As an alternative to antibiotics, nanoparticles (NPs) are widely used in antimicrobial applications, including metal-based NPs, carbon-based NPs and polymer-based NPs [6]. Of these, silver nanoparticles (Ag NPs) exhibit potent antimicrobial activity and a broad biocidal spectrum, effective against Gram-positive and Gram-negative bacteria, fungi, viruses and mycobacteria [7]. However, their inherent instability limits their clinical application, as Ag NPs readily form aggregates under physiological conditions because of inorganic salts and proteins [8]. In our previous reports, we modified Ag NPs with biodegradable polymer poly(lactic-co-glycolic) (PLGA) to improve the stability of Ag NPs [8].

PLGA is a block co-polymer composed of lactate and glycolate that has been approved by the FDA [9]. PLGA has been widely used for the controlled and targeted delivery of therapeutics because of its high biocompatibility and biodegradability [10]. In our previous report, we successfully encapsulated Ag NPs within PLGA NPs, which enhanced their stability under physiological conditions [8]. However, unmodified PLGA NPs possess certain inherent limitations, most notably their negative surface charge, which hampers efficient cellular uptake [10]. Polyethylene imine (PEI), a commercially available cationic polyamine, is a successful and widely studied cationic polymer, and surface modification of PLGA NPs with PEI was shown to improve cellular uptake of these modified NPs [11].

Bacterial membrane vesicles (MVs) are nano-sized vesicles released from bacteria that contain lipids, bioactive proteins, nucleic acids and metabolites and play important roles in microbial physiology [12]. MVs from Gram-negative bacteria (such as *S. Typhimurium*) contain lipopolysaccharide (LPS), which is well known to stimulate inflammatory reactions [13,14]. A previous report from Chen et al. showed that removing LPS from MVs reduced cellular uptake by macrophages [15]. In addition, MVs from *S. Typhimurium* were observed in the vicinity of SCV membranes, and they could be transported across the SCV membrane to the macrophage cytoplasm [16]. Recently, Wu et al. reported that modifying PLGA NPs with MVs from *Escherichia coli* using a microfluid method facilitated active NP uptake by macrophages and increased the M1 phenotype (pro-inflammatory) of macrophages [17]. Methods of extrusion, sonication, electroporation and microfluidics have been reported to facilitate combining NPs with membranes (not limited to MVs) [18]. Of these methods, extrusion and sonication are the most frequently used. Compared with the extrusion method, the sonication method is simple and overcomes problems with large-scale production.

In this study, we prepared Ag-loaded PLGA NPs, as described in our previous work, and modified their surface charge to positive by incorporating PEI to yield Ag-PP NPs. MVs were isolated from *S. Typhimurium* culture supernatants and combined with Ag-PP NPs using a sonication-based fusion method, resulting in MV-coated nanoparticles (Ag-PP@MV NPs). Microscopic observations and protein analysis confirmed the successful modification of Ag-PP NPs with MVs. Compared with Ag-PP NPs, Ag-PP@MV NPs exhibited enhanced cellular uptake by macrophages and demonstrated improved antibacterial activity against intracellular *S. Typhimurium*.

## 2. Materials and Methods

### 2.1. Chemicals

Silver nitrate (0.1 M), poly(vinyl alcohol), poly(*p*-styrenesulfonic acid) solution (MW 70,000), chloroform and 2-mercaptoethanol were purchased from FUJIFILM Wako Pure Chemical Corporation (Osaka, Japan). Trisodium citrate, sodium borohydride, *L*(+)-ascorbic acid, tryptone, dried yeast extract, sodium chloride, agar powder and the protein assay BCA kit were purchased from Nacalai Tesque (Kyoto, Japan). Disulfiram (DSF) was purchased from Tokyo Chemical Industries (Tokyo, Japan). SH-PEG-COOH (5000 Da) was purchased from Biopharma PEG Scientific Inc. (Watertown, MA, USA). The fluorescent agent of 1,1′-diotadecyl-3,3,3′,3′-tetramethylindocarbocyanine perchlorate (DiI) was purchased from MedChemExpress (Monmouth Junction, NJ, USA). Polyacrylamide gradient gels (5.0–20%) and AE-1360 EzStarin Silver were purchased from ATTO Corporation (Tokyo, Japan). Rapid flow filter units (0.45 µm) were purchased from Thermo Fisher Scientific (Waltham, MA, USA). Cell culture plates were purchased from Costar, Corning (Corning, NY, USA).

### 2.2. Preparation of NPs

Silver nanoplates (Ag NPLs) were prepared via a two-step process following previously reported methods [8]. A seed solution of Ag nanoparticles was first prepared by mixing 20 mL of 2.5 mM trisodium citrate, 1 mL of 0.5 g/L polystyrene sulfonate, 1.2 mL of 10 mM NaBH_4_ and 50 mL of 0.5 mM silver nitrate in a flask. This solution was mixed for 60 min at 30 °C. Next, 100 mL of water and 2.3 mL of 10 mM ascorbic acid were added to 1 mL of the above seed solution. Then, 60 mL of 0.5 mM silver nitrate and 10 mL of 25 mM sodium citrate were added, and the solution was kept at 30 °C for 100 h. After centrifugation (12,000× *g*, 25 °C, 10 min), the Ag NPLs were obtained. Since the bare Ag NPLs are unstable and typically undergo aggregation, surface modification with PEG was further performed. However, hydrophilic PEG-Ag NPLs are not suitable for encapsulating the Ag NPLs into PLGA NPs. Thus, to prepare the hydrophobic Ag NPLs, the PEG-Ag NPLs were treated with DSF, as reported previously [8].

PLGA (6 mg/mL) and DSF (1.8 mg/mL) were dissolved in chloroform, and the PEG-Ag NPLs aqueous solution was added. The double-layer solution was stirred at 1000 rpm until the Ag NPLs were transferred to the chloroform layer. The hydrophobic Ag NPLs were then encapsulated into PLGA NPs using ultrasonication at 60% amplitude for 6 min (pulse on 50 s, pulse off 10 s) with a micro sonication homogenizer (Qsonica, Q125, Newtown, CT, USA). After sonication, chloroform in the solution was volatilized by stirring for 3 h to obtain a water dispersion. The solution was centrifuged (1500× *g*, 25 °C, 5 min) to precipitate large particles, and the supernatant was further centrifuged (12,000× *g*, 25 °C, 10 min) to remove PVA. The Ag-loaded PLGA (Ag-P) NPs were obtained.

To prepare MVs, *Salmonella enterica* serovar Typhimurium (*S. Typhimurium*; LT-2 strain) was incubated in 2 L of liquid LB medium at 37 °C until reaching an OD_620_ of 1.0. The cell suspension was then filtered via a Rapid Flow Filter unit (0.45 µm) and concentrated using 100K ultra centrifugal filters (Merck Millipore, Billerica, MA, USA). MVs were then collected by ultracentrifugation at 50,000× rpm (126,000× *g*) for 1 h with an ultracentrifuge (Himac CS 100 GXII, Hitachi Koki Co., Ltd., Tokyo, Japan), dispersed in phosphate-buffered saline (PBS) and stored at −80 °C.

To facilitate MV coating, the surface charge of PLGA NPs was modified by incubating with 0.01% PEI solution for 10 min. The resulting PEI-coated Ag-PLGA NPs (Ag-PP NPs) were collected by centrifugation at 12,000× *g* and 25 °C for 10 min. Equal volumes of Ag-PP NPs (20 ppm, as Ag concentration) and MVs (100 µg/mL, as protein concentration) were mixed under sonication with the same micro-sonication homogenizer described above at 20% amplitude for 2 min (pulse on 10 s, pulse off 10 s). The MV-coated Ag-PP NPs (Ag-PP@MV NPs) were recovered by centrifugation at 12,000× *g* and 25 °C for 10 min.

### 2.3. Characterization of NPs

The morphology of Ag NPLs and Ag-PP@MVs NPs was examined using a transmission electron microscope (TEM) (HT7700, Hitachi, Tokyo, Japan) operated at 80 kV. Samples were negatively stained with 1.5% uranyl acetate and vacuum-dried at room temperature for 24 h before imaging. NP size and surface charge (zeta potential) were measured by dynamic light scattering (DLS) using a Zetasizer Nano ZS (Malvern Instruments, Malvern, UK). The size distribution and particle concentration of isolated MVs were analyzed by nanoparticle tracking analysis (NTA) using a NanoSight NS300 (Quantum Design, Tokyo, Japan). Elemental composition of the MVs and Ag-PP@MVs NPs was assessed by energy dispersive X-ray (EDX) spectroscopy using a FEI TECNAI F20 microscope (Thermo Fischer Scientific, Waltham, MA, USA).

Sodium dodecyl sulfate-polyacrylamide gel electrophoresis (SDS-PAGE) analysis of MVs and Ag-PP@MVs was performed to confirm successful MV coating on the PLGA-PEI NPs. Samples were heated at 95 °C for 5 min and loaded onto 5–20% gradient polyacrylamide gels. Protein bands were visualized using silver nitrate staining.

### 2.4. Silver Ions Released from NPs

Ag-PP NPs and Ag-PP@MV NPs with a concentration of 5 ppm were prepared. The NPs were centrifugated and dispersed in 3 mL of PBS. The solutions were allowed to incubate at 37 °C for 2 h. After centrifugation of NPs, Ag^+^ ions remaining in the supernatant were quantified by inductively coupled plasma-optical emission spectroscopy (ICP-OES; iCAP 7000 series, Thermo Fisher Scientific).

### 2.5. Cytotoxicity Evaluation

The cytotoxicity of the NPs to macrophage RAW 264.7 cells was examined using the Cell Counting Kit-8 Assay (Dojindo Laboratories, Kumamoto, Japan). Briefly, RAW 264.7 cells were seeded into 96-well plates at 4 × 10^4^ cells/well and incubated overnight in D-MEM containing 10% FBS and 1% penicillin–streptomycin solution. Ag-PP NPs and Ag-PP@MV NPs were then added to the cells at concentrations of 3 and 5 ppm. After incubation for 2 and 3 h, the plates were washed with PBS, then incubated with 100 μL of fresh D-MEM containing 10% FBS and 10 μL of CCK-8 solution, separately. The absorbance at 450 nm was measured using a microplate reader (TECAN Infinite 200pro M Nano, Tecan, Männedorf, Switzerland).

### 2.6. Cellular Uptake of NPs by RAW 264.7 Cells

The cellular uptake of Ag-PP NPs and Ag-PP@MV NPs was evaluated by seeding RAW 264.7 cells in 6-well plates at 2 × 10^5^ cells/well and incubating overnight in D-MEM containing 10% FBS and 1% penicillin–streptomycin. Ag-PP NPs and Ag-PP@MV NPs were then added at a final concentration of 3 ppm, followed by incubation for 2 h. After treatment, the cells were harvested and dissolved in 1 M nitric acid. The solution was heat-dried, and the resulting residue was re-dissolved in 0.1 M nitric acid. The intracellular concentration of Ag^+^ ions was quantified by ICP-OES.

### 2.7. Antibacterial Activity of NPs Against Intracellular Bacteria

The antibacterial activity against intracellular bacteria was evaluated according to our previous report [8], with minor modifications. Briefly, RAW 264.7 cells were seeded into 24-well plates and incubated overnight. The cells were then infected with *S. Typhimurium* at a multiplicity of infection (MOI) of 100. After incubation for 1 h, planktonic bacteria were gently removed by washing with PBS and further incubated with fresh D-MEM containing 10% FBS and 100 μg/mL gentamycin for 2 h to eliminate extracellular bacteria. After washing with PBS, NPs in D-MEM containing 20 μg/mL gentamycin and 2% FBS were added to the cells. After 2 h incubation, cells were gently washed with PBS and lysed using PBS containing 0.5% sodium deoxycholate. The lysates were serially diluted, and 100 μL of each solution was plated onto LB/ampicillin agar plates. Intracellular bacteria survival was assessed by colony-forming unit (CFU) counts.

Green fluorescent protein (GFP)-expressing *S. Typhimurium* and DiI-labeled NPs were used to visualize the intracellular localization of bacteria and NPs. The cell treatments mentioned above were performed after infection of cells with GFP-expressing *S. Typhimurium*. DiI-labeled NPs were then added to cells and incubated for 2 h. Cells were washed with PBS and observed under a fluorescence microscope (Zeiss, Oberkochen, Germany).

### 2.8. Statistical Analysis

Experiments were performed in triplicate, and data are presented as the mean ± standard deviation (SD). Data were analyzed using Student’s *t*-test (GraphPad Prism, Version 10). A difference was considered statistically significant if the *p*-value was <0.05.

## 3. Results and Discussion

### 3.1. Characterization of NPs

**Figure 1:**
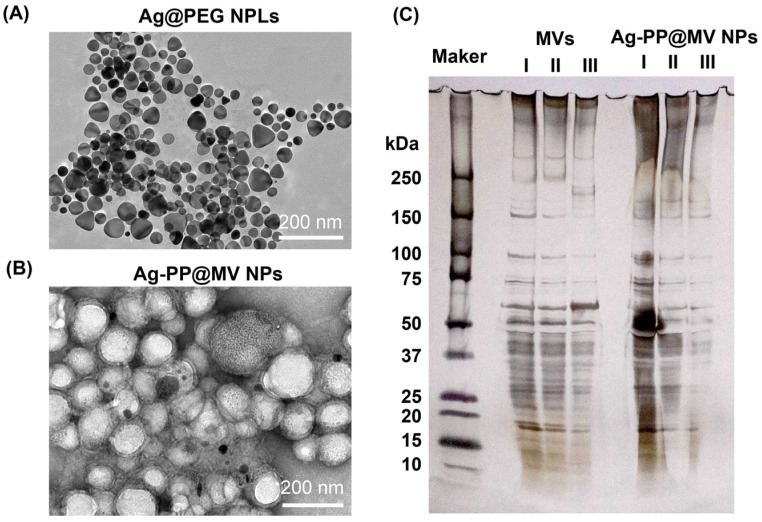
Transmission electron microscopy images of Ag@PEG NPLs (**A**) and Ag-PP@MV NPs (**B**). Protein profile of MVs and Ag-PP@MV NPs separated by SDS-PAGE and visualized using silver staining (**C**) (MVs lanes I–III: 7.5, 5.0 and 2.5 µg of MVs; Ag-PP@MV NPs lanes I–III: 2.0, 1.0 and 0.5 µg of Ag).

Triangular Ag NPLs were synthesized using a previously reported two-step process [8]. As shown in Figure 1A, TEM images confirmed the formation of plate-like morphologies. Due to the fact that bare Ag NPLs were aggregated easily, we showed the images of Ag NPLs with PEG modification (Ag@PEG NPLs). DLS analysis revealed two distinct size distributions at 4.0 and 56.8 nm (Table 1), corresponding to the vertical thickness and lateral dimensions of the nanoplates, respectively [19]. Treatment of the Ag@PEG NPLs with the hydrophobic agent DSF, which contains a disulfide bond, rendered the surface hydrophobic. The hydrophobic Ag NPLs were encapsulated in PLGA NPs using an emulsion-based method. The resulting Ag NPL-loaded PLGA NPs (Ag-P NPs) exhibited a single size distribution centered at 245.9 nm and possessed a negatively charged surface (Table 1). Following surface modification with PEI, the size of Ag-PP NPs remained essentially unchanged, but the surface charge shifted to a positive value. DLS analysis showed that MVs exhibited a single size distribution centered at 132.9 nm and a negatively charged surface. After modifying Ag-PP NPs with MVs using the sonication method, the resulting Ag-PP@MVs NPs displayed a net negative surface charge, consistent with successful MV coating. TEM images (Figure 1B) revealed a visible coating layer surrounding the PLGA NPs, and the observed particle size of Ag-PP@MV NPs ranged from approximately 150 to 200 nm. The NP sizes measured by TEM were smaller than those determined by DLS analysis, which can be attributed to DLS measuring the hydrodynamic diameter, a measurement biased by the higher scattering intensity of larger particles [20]. Zeta potential analysis and TEM imaging confirmed that Ag-PP NPs were successfully modified with MVs.

SDS-PAGE and silver staining were performed to confirm the retention of membrane proteins from native MVs within Ag-PP@MV NPs. As shown in Figure 1C, similar protein profiles from MVs and Ag-PP@MV NPs were observed, showing that the modification process (including sonication) did not significantly change the protein content of MVs. Lanes representing MVs I–III were loaded with 7.5, 5.0 and 2.5 µg of MVs (as protein weight), whereas lanes Ag-PP@MV NPs I–III were loaded with 2.0, 1.0 and 0.5 µg of NPs (as Ag weight). In our previous study, Ag NPL loading in PLGA NPs was calculated to be approximately 5% [8]. Therefore, according to the band densities, we postulated that approximately 20 µg of NPs was combined with 5 µg of MVs (as protein weight). We also attempted to modify PLGA NPs with MVs using the same sonication method; however, PLGA NPs were not modified with MVs by the sonication method because both species have a negative surface charge (no bands appeared in lanes loaded with Ag-P@MVs NPs).

### 3.2. Morphology and Elemental Composition Analysis

**Figure 2:**
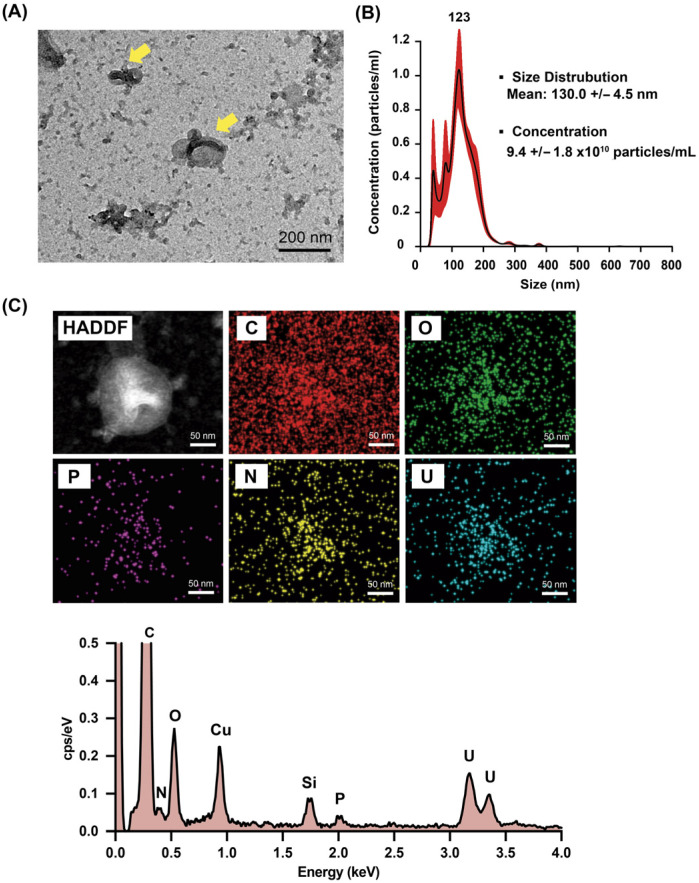
TEM image (**A**), nanosight analysis (**B**), energy dispersive X-ray spectroscopy element mapping and spectrum of MVs (**C**).

In this study, we also evaluated the morphology and elemental composition of MVs using TEM and EDS. Uranyl acetate was used to stain MVs because uranyl ions bind to proteins and lipids, thereby improving the electron density [21]. Vesicles with sizes of ~200 nm and the typical cup-shaped morphology of MVs were observed by TEM (Figure 2A), which is similar to other extracellular vesicles (EVs) [22]. We further quantified MVs by NTA. As shown in Figure 2B, MVs displayed a mean particle size of 130 nm and a mean particle concentration of 9.4 × 10^10^ particles/mL. The EDS elemental maps and spectrum (Figure 2C) showed that MVs contain carbon (C), oxygen (O), nitrogen (N) and phosphorus (P) as typical elements of lipid vesicle membranes [23]. There are only a few element analysis reports of EVs by EDS, such as those for mesenchymal stem cell-derived EVs [24] and B-cell-derived EVs [23]. Mesenchymal stem cell-derived EVs showed a rich calcium content, whereas B-cell-derived EVs showed a similar content to our MVs.

**Figure 3:**
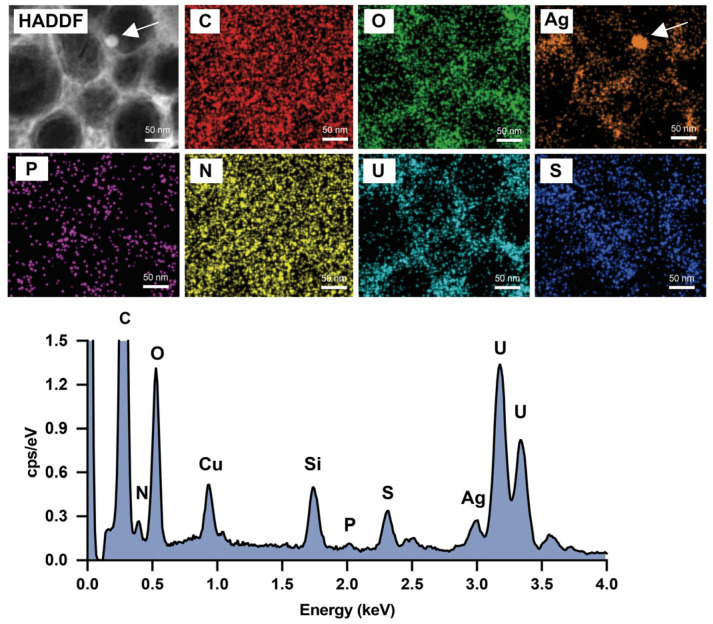
Scanning-TEM image, energy dispersive X-ray spectroscopy element mapping and spectrum of Ag-PP@MVs. White arrows indicate hydrophilic Ag NPLs.

We next analyzed the elemental composition of Ag-PP@MV NPs using EDS. As shown in Figure 3, the elemental maps and spectrum revealed that Ag-PP@MV NPs contain C, O, N, Ag, sulfur (S) and a small amount of P. Compared with the element profile of native MVs, the additional detection of Ag and S confirmed the successful incorporation of Ag into the Ag-PP@MV NPs. However, the spatial distributions of Ag and S did not overlap entirely. We propose that the resulting Ag–S NPLs were more efficiently encapsulated within PLGA NPs because of their smaller size and hydrophobic surface. In contrast, unmodified Ag NPLs (which do not contain S and are marked by white arrows in Figure 3) were larger and more hydrophilic, making them less likely to be encapsulated and more likely to be adsorbed onto or trapped by the PLGA matrix. These observations are consistent with a previous report showing that Ag–S NPs exhibit low aqueous solubility (i.e., low hydrophilicity) and are unstable under light irradiation (easily degraded to smaller NPs) [25].

### 3.3. Silver Ions Released from NPs and Cytotoxicity Evaluation

**Figure 4:**
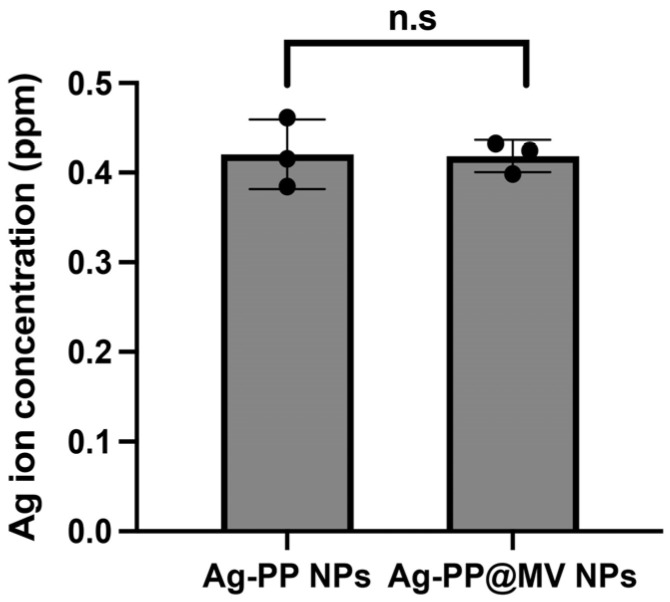
Silver ions released from Ag-PP and Ag-PP@MV NPs in PBS at 37 °C for 2 h (n.s., not significant). Data represent the mean ± SEM of triplicates.

To assess Ag^+^ ion release, Ag-PP and Ag-PP@MV NPs (5 ppm) were incubated in PBS at 37 °C for 2 h. The NPs were harvested by centrifugation, and the Ag^+^ ions remaining in the supernatant were quantified by ICP-OES. Both NP formulations showed comparable Ag^+^ ion release profiles of 0.4 ppm at 2 h (approximately 8% of total Ag) (Figure 4). This result indicated that the released Ag^+^ ions from Ag-PP@MV NPs were not captured by surface-modified MVs.

**Figure 5:**
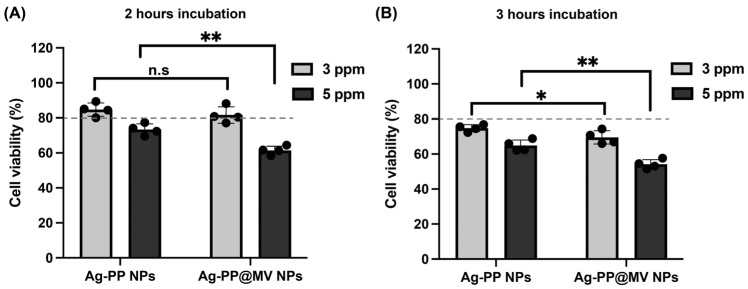
Cytotoxicity evaluation of Ag-PP and Ag-PP@MV NPs at 3 and 5 ppm after incubation with RAW264.7 cells for 2 h (**A**) and 3 h (**B**). (* *p* ≤ 0.05, ** *p* ≤ 0.01; n.s., not significant). Data represent the mean ± SEM of four replicates. Gray dotted lines indicate 80% viability threshold.

Further, Ag^+^ ions can be cytotoxic to mammalian cells. Similarly to the antibacterial mechanism of Ag NPs, Ag NPs or released Ag^+^ ions can cause oxidative stress via damage complex biomolecules, including DNA, enzymes, proteins and cell membranes [5]. Murine macrophage RAW 264.7 cells were used as a model host for intracellular bacterial infection. We evaluated the cytotoxicity of Ag-PP and Ag-PP@MV NP at concentrations of 3 and 5 ppm after incubation with RAW 264.7 cells for 2 and 3 h, respectively. As shown in Figure 5A, after 2 h of incubation with RAW 264.7 cells, Ag-PP and Ag-PP@MV NPs at 3 ppm did not show cytotoxicity (viability > 80%), whereas Ag-PP@MV NPs at 5 ppm showed higher cytotoxicity (viability at 61%) than Ag-PP NPs (viability at 73%). After 3 h of incubation with RAW 264.7 cells, both Ag-PP NPs and Ag-PP@MV NP showed high cytotoxicity, even at 3 ppm. Therefore, in further experiments, we used Ag-PP and Ag-PP@MV NPs at 3 ppm, and incubated NPs with cells for 2 h.

### 3.4. Cellular Uptake of NPs by Macrophages and Antibacterial Activity of NPs Against Intracellular Bacteria

**Figure 6:**
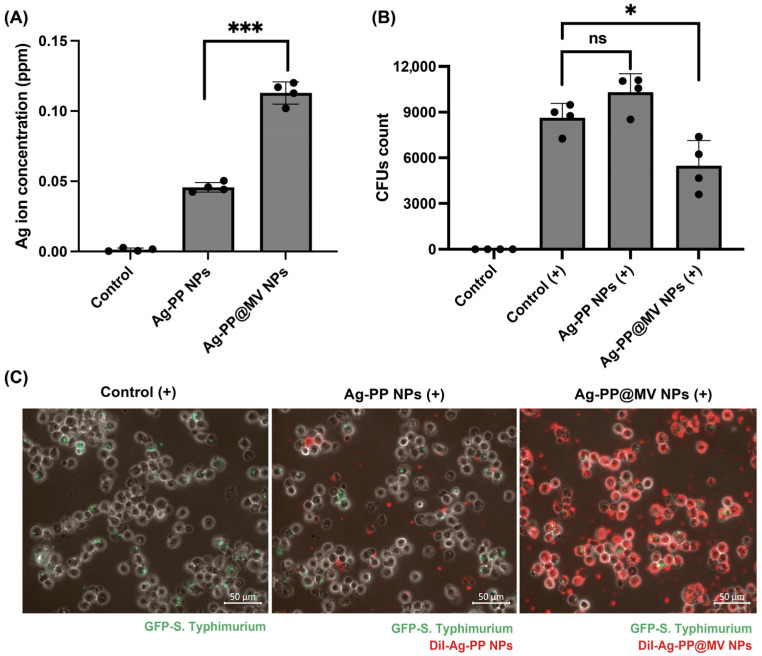
Cellular uptake of Ag-PP and Ag-PP@MV NPs by RAW 264.7 cells (**A**). Intracellular antibacterial activity (**B**) and fluorescence images (**C**) of the NPs against intracellular *S. Typhimurium* (* *p* ≤ 0.05, *** *p* ≤ 0.001; n.s., not significant). Data represent the mean ± SEM of four replicates.

*S. Typhimurium* is an intracellular microorganism capable of surviving and replicating in macrophages. The cellular uptake of Ag was quantified by measuring the intracellular Ag^+^ levels in RAW 264.7 cells following exposure to Ag-PP and Ag-PP@MV NPs using ICP-OES. As shown in Figure 6A, Ag^+^ ion accumulation was significantly higher in cells treated with Ag-PP@MV NPs (0.12 ppm, approximately 4% of Ag), compared with those treated with Ag-PP NPs (0.05 ppm, approximately 1.7% of Ag). MVs play crucial roles in bacterial pathogenesis by promoting colonization, transmitting virulence factors into host cells and modulating host cell responses [26]. Notably, Alaniz et al. reported that *S. Typhimurium* MVs efficiently activate macrophages and dendritic cells, enhancing the production of pro-inflammatory mediators [14]. Based on these observations, we hypothesized that MV modification, which contains LPS and bioactive proteins, may enhance NP uptake by macrophages through immune activation. This concept may also explain the higher cytotoxicity of Ag-PP@MV NPs compared with Ag-PP NPs, despite their similar Ag^+^ release profiles. Therefore, these findings suggest that cellular uptake is a critical determinant of NP-associated toxicity in mammalian cells.

We then assessed the intracellular antibacterial activity of the NPs against *S. Typhimurium* using a gentamicin protection assay, and quantified viable bacteria by the CFU method. Treatment with Ag-PP NP did not reduce the number of intracellular *S. Typhimurium* (Figure 6B). In contrast, treatment with Ag-PP@MV NP resulted in an ~20% reduction in intracellular bacterial load. Although this reduction was modest, it suggests that MV modification may enhance the intracellular antibacterial efficacy of Ag NPs. Fluorescence microscopy revealed that DiI-labeled Ag-PP@MV NPs were more efficiently internalized by RAW 264.7 cells than DiI-labeled Ag-PP NPs, which is consistent with the ICP-OES results (Figure 6C). However, selective uptake of Ag-PP@MV NPs by only infected RAW 264.7 cells was not observed. Despite the enhanced cellular uptake, this lack of specificity limits the overall intracellular antibacterial effect.

These findings indicate that although MV modification improves NP uptake by macrophages, further optimization, such as engineering selective targeting toward infected macrophages, is required to enhance intracellular antibacterial efficacy. In addition, we considered the following as potential reasons for this limited efficacy: Ag-PP@MVs did not sufficiently co-localize with intracellular bacteria in SCVs or the amount of released Ag ions was insufficient. As described in the Introduction, MVs from *S. Typhimurium* have been observed in the vicinity of SCV membranes, and they could be transported across the SCV membrane to the cytoplasm [16]. Therefore, Ag-PP@MVs have the potential to co-localize with intracellular bacteria in SCVs. However, in this study, to avoid damaging the host cells, we limited the treatment time of RAW 264.7 cells and concentration of NPs, which likely led to insufficient delivery of NPs and Ag ions into SCVs. In addition, the release of Ag ions from Ag-PP@MV NPs under acidic conditions (pH 5.0, mimicking SCVs) may also be an important factor. Gnopo et al. reported that MVs from *E. coli* form aggregates and undergo fusion under acidic conditions [27]. However, MVs from *S. Typhimurium* should be stable under acidic conditions because they contain pH-responsive proteins [28] and can be transported across the SCV membrane to the cytoplasm, as described above [16]. Similarly, we suggest that Ag-PP@MVs would not form aggregates, and would release Ag ions under both acidic and neutral conditions. Above all, developing strategies for selective delivery to infected *S. Typhimurium* cells, increasing the amount of NPs in SCVs, and increasing the amount of Ag ions released in SCVs represent the next promising steps in advancing this therapeutic approach.

## 4. Conclusions

In this study, we successfully modified Ag-loaded PLGA NPs with bacterial MVs derived from *S. Typhimurium* (Ag-PP@MV NPs) using the sonication method. The resulting Ag-PP@MV NPs retained a protein profile comparable to native MVs and exhibited sustained Ag^+^ ion release to ensure antibacterial activity against planktonic *S. Typhimurium*. Notably, Ag-PP@MV NPs were more actively taken up by RAW 264.7 cells and showed modest antibacterial activity against intracellular *S. Typhimurium* compared with Ag-PP NPs. Although the intracellular antibacterial activity requires further improvement, MV modification of NPs represents a promising strategy to enhance Ag^+^ delivery to macrophages and, thus, the treatment of intractable bacterial infections.

## Figures and Tables

**Table 1:** Size and zeta potential of nanoparticles.

Nanoparticles	Size (nm)	Zeta Potential (mV)
Ag@PEG NPLs	4.0; 56.8	−27.8
MVs	132.9	−25.3
Ag-P NPs	245.9	−27.6
Ag-PP NPs	218.5	+26.8
Ag-PP@MVs NPs	250.1	−16.0

## Data Availability

The original contributions presented in this study are included in the article. Further inquiries can be directed to the corresponding author.
